# Stoichiometric analysis of protein complexes by cell fusion and single molecule imaging

**DOI:** 10.1038/s41598-020-71630-6

**Published:** 2020-09-10

**Authors:** Avtar Singh, Alexander L. Van Slyke, Maria Sirenko, Alexander Song, Paul J. Kammermeier, Warren R. Zipfel

**Affiliations:** 1 Applied and Engineering Physics, Cornell University, Ithaca, NY 14853 USA; 2 Graduate Field of Biophysics, Cornell University, Ithaca, NY 14853 USA; 3 Department of Biological Engineering, Cornell University, Ithaca, NY 14853 USA; 4 Department of Pharmacology and Physiology, University of Rochester Medical Center, Rochester, NY USA; 5 Meinig School of Biomedical Engineering, Cornell University, 101 Weill Hall, Ithaca, NY 14853 USA; 6 Present Address: Broad Institute, Cambridge, MA 02142 USA; 7 Present Address: University of Pennsylvania, Philadelphia, PA 19104 USA; 8 Present Address: Memorial Sloan Kettering Cancer Center, New York, NY 10065 USA; 9 Present Address: Max Planck Institute for Intelligent Systems, Stuttgart, Germany

**Keywords:** Biophysics, Biological fluorescence, Biophysical methods, Microscopy

## Abstract

The composition, stoichiometry and interactions of supramolecular protein complexes are a critical determinant of biological function. Several techniques have been developed to study molecular interactions and quantify subunit stoichiometry at the single molecule level. However, these typically require artificially low expression levels or detergent isolation to achieve the low fluorophore concentrations required for single molecule imaging, both of which may bias native subunit interactions. Here we present an alternative approach where protein complexes are assembled at physiological concentrations and subsequently diluted in situ for single-molecule level observations while preserving them in a near-native cellular environment. We show that coupling this dilution strategy with fluorescence correlation spectroscopy permits quantitative assessment of cytoplasmic oligomerization, while stepwise photobleaching and single molecule colocalization may be used to study the subunit stoichiometry of membrane receptors. Single protein recovery after dilution (SPReAD) is a simple and versatile means of extending the concentration range of single molecule measurements into the cellular regime while minimizing potential artifacts and perturbations of protein complex stoichiometry.

## Introduction

Dynamic networks of protein interactions underlie much of cell biology. A key goal of biomedical science is to understand the nature of these interactions and how they change in response to various extracellular cues. The native subunit stoichiometry of protein complexes often plays an important role in determining and regulating a protein’s function. Screening methods, such as yeast-two hybrid analysis or phage display, are useful for identifying potential binding partners in a high-throughput manner, but generally ignore biological context^1^. Ensemble approaches that rely on co-immunoprecipitation or fluorescence spectroscopy can more accurately capture interactions within the cellular environment and are used to examine changes that occur in response to external stimuli^1,2^. However, these bulk ensemble averaged measurements yield little information about the stoichiometry of subunits within complexes. Single molecule methods have the sensitivity to probe single protein complexes and quantitatively report on their individual architectures, further enabling the detection of critical subpopulations or heterogeneities.

Early uses of single-molecule fluorescence for subunit counting relied on artificially low expression levels in order to resolve individual protein complexes^3^. However, non-physiological concentrations during assembly can shift binding equilibria and alter normal stoichiometry. Alternatively, a single-molecule pull-down (SiMPull) approach has been developed so that complexes can be assembled at native expression levels, extracted into a cell lysate, and then captured on an antibody-coated slide for single-molecule imaging^4^. Antibody concentrations and lysate dilutions can be tuned to maintain single-molecule resolution without compromising intracellular assembly conditions. Although SiMPull has been used to measure the subunit stoichiometry of membrane receptors, mitochondrial proteins, virus replication initiation, nuclear export complexes, and signaling complexes, the use of detergents for isolation and subsequent wash steps has been noted to affect the integrity of some macromolecular assemblies, particularly of membrane receptors, and therefore the physiological relevance of stoichiometry data^5–7^.

Here, we introduce a simple detergent-free method to examine single protein complexes assembled at physiological concentrations in a near-native environment. Two cell populations—one containing a protein complex of interest and the other lacking it—are co-plated on a coverslip and fused into large syncytia. Protein diffusion within these syncytia results in a dilution of labelled complexes permitting their examination at reduced concentrations. Dilution factors may be controlled by varying the co-plating ratio and can be made sufficiently high to resolve single membrane protein complexes in TIRF for stepwise photobleaching and brightness analysis, 2D membrane fluorescence correlation spectroscopy (FCS), two-color single molecule colocalization or single molecule FRET experiments. Cytosolic proteins are diluted as well and high-quality in vivo FCS and FCCS data can be obtained using the method. We call our approach Single Protein Recovery After Dilution (SPReAD), as it yields concentrations suitable for single molecule imaging after physiological oligomer assembly.

## Results

### Formation of large syncytia using an inducible vesicular stomatitis virus G protein expressing cell line

**Figure 1:**
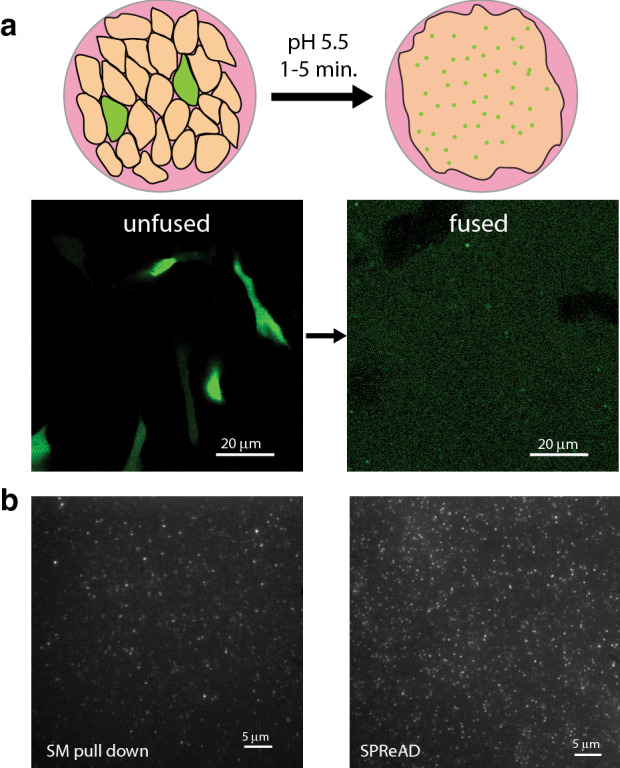
Single Protein Recovery After Dilution (SPReAD) for single molecule measurements. (**a**) Cells expressing a labeled protein-of-interest are co-plated with a stable U2OS cell line which express vesicular stomatitis virus (VSVG) after doxycycline activation. A brief incubation in low pH (5.5) buffer initiates membrane fusion, after which protein complexes diffuse out of their parent cells into the larger syncytium [mNeonGreen in (**a**)]. (**b**) mNeonGreen-ADRβ2 (mNG-ADRβ2) protein complexes prepared for single molecule imaging by detergent isolation and biotin-streptavidin pull-down (left) and mNG-ADRβ2 protein complexes in the syncytium membrane after VSVG-mediated fusion (right).

A stable cell line with conditional expression of vesicular stomatitis virus G protein (VSVG) was created and used to initiate controlled cell fusion between the VSVG expressing cell line and cells expressing a labeled protein of interest. VSVG is a well-characterized fusogen that can be reversibly activated by a short pH drop^8^. To dilute protein complexes for stoichiometric analysis, doxycycline-inducible VSVG-expressing U2OS cells were mixed with cells expressing the target protein typically at a 10:1 ratio (VSVG cells to target cells) and incubated at pH 5.5 for 5 min. After activating VSVG in a confluent monolayer of the mixed cell culture, we observed rapid formation (< 1 h) of massive syncytia and diffusion of fluorescent proteins such as mNeonGreen (Fig. 1a) producing a uniform distribution in the cytosol. Fusion with cells expressing membrane targeted proteins (mNeonGreen-beta-2-adrenergic receptor in this case) results in syncytia with punctate spots (Fig. 1b, right), resembling what is found using single molecule pull-downs of detergent-isolated proteins from the same cells (Fig. 1b, left). This suggests that substantial dilution factors may be attained in time intervals comparable to handling times for cell lysate preparation, implying that the two approaches have similar bounds for detecting transient, non-covalent oligomerization. However, SPReAD has the advantage of not requiring a detergent isolation step, which could disrupt non-covalent interactions and/or perturb protein function^5–7^, and the initial intracellular complex formation is carried out under normal cellular conditions before cell fusion.

The dynamics of cell fusion and long-term viability of syncytia was visualized using time-lapse bright-field imaging (SI Appendix Fig. Fig. S1a) which showed that fusion was accompanied by a loss of cell boundaries approximately 40 min after pH drop. For the next ~ 4–5 h, the syncytium remained bound to the coverslip and displayed few morphological changes. Thereafter, adhesion was slowly lost over the course of 12 h and, at 20 h, concerted cell death was observed. This suggests there is a 4 to 5-h period during which cells are fused, but otherwise minimally perturbed. Cells may be imaged live during this window or fixed for later observation. Local spreading by diffusion of both cytosolic and membrane proteins occurs rapidly (SI Appendix Fig. S1b, c and d) and single molecule compatible levels are reached in 20–30 min.

Large-scale fusion was possible in all mammalian cell lines tested as expected due to the broad tropism of VSVG (SI Appendix, Fig. S2). This provides an additional means of experimental control by which background protein factors can be tuned by choice of cell type.

The formation of syncytia is a key step in the development of various mammalian tissues, including bone, muscle and placenta^9^. In these cases, cell fusion is well regulated and part of the normal developmental program. Cell fusion can also play a role in the progression of disease. Many enveloped viruses trigger fusion between an infected cell and its neighbors, resulting in new and abnormal hybrids. Accidental cell fusion, both due to viral infection and otherwise, has also been implicated in cancer, where polyploid cells display high levels of chromosomal instability and may acquire tumorigenic phenotype^10^. These natural examples of cell fusion highlight syncytia as a biologically relevant state. Although different from the original unfused cells, we believe that engineered syncytia will preserve pre-assembled protein complexes better than alternative approaches (e.g., detergent isolation) and are a promising system for single-molecule stoichiometry studies.

### Dilution of labelled cytosolic proteins by cell fusion improves in vivo fluorescence correlation spectroscopy

Since its invention in the 1970s, fluorescence correlation spectroscopy (FCS) has become a valuable tool for investigation of molecular transport and interactions^11^. Autocorrelation analysis provides information about diffusion, per-particle brightness and local concentrations, while two-color fluorescence cross-correlation spectroscopy (FCCS) can probe molecular associations^12^. FCS and FCCS can be used inside living cells, but cellular proteins are typically expressed at intracellular concentrations outside of the working range for FCS studies. Furthermore, standard FCS and FCCS fitting models assume an infinite pool of diffusive species such that molecular motions are unconstrained and photobleaching is inconsequential. However, this is hardly the case within the cellular environment and these assumptions are known to lead to artifacts^13^. Cell fusion is a promising means to address both of these limitations as concentrations can be tuned to fall inside the optimal range for FCS, and the relatively large size of the syncytium serves to alleviate the effects of constrained motion and photobleaching that can occur during live-cell FCS.

For cell fusion to function as a dilution strategy, protein complexes must be sufficiently mobile to diffuse out of their parent cells into the larger syncytium. Proteins confined to specific organelles or stably tethered to cytoskeletal components may fail to satisfy this criterion; however, many transcription factors and signaling complexes have mobile cytoplasmic fractions and are candidates for cell fusion-based dilution and single molecule analysis. The kinetics of syncytium formation and protein mobility determine the optimal timeframes for imaging and fixation after fusion is initiated. Time-lapse imaging revealed that membrane fusion was immediate and synchronized across the imaging dish, with cytosolic proteins beginning to escape their parent cells within 2 min of pH drop (SI Appendix Fig. S3). The initially heterogeneous fluorescence distribution was continually reshaped by diffusion until reaching a uniform steady-state level after ~ 30 min. The equilibration time depends on the size of protein complexes being studied, their interactions with static cellular components, experimental conditions and the ratio of expressing and non-expressing cells. Overall, the kinetics of cell fusion and protein redistribution provide two possible modes of measurement. Measurements made in the non-equilibrium mode, prior to equilibration of the protein distribution, will most accurately report on the stoichiometry of weakly interacting protein complexes because assemblies have less time to dissociate before recording. However, concentration measurements at this stage will be heterogeneous across the imaging dish. In contrast, equilibrium mode measurements can be used to back-calculate the average intracellular concentrations prior to cell fusion (based on a known co-plating ratio), but may provide less accurate stoichiometric measurement of complexes with the fastest dissociation rates. Overall, this flexibility renders SPReAD as a versatile method for quantification of both oligomeric state and cellular expression levels.

To determine the range of dilutions possible, non-fluorescent VSVG-expressing cells were mixed with cells stably expressing mNeonGreen (mNG) at various co-plating ratios. After fusion, the fluorescence signal per unit area dropped in proportion to the co-plating ratio (SI Appendix, Fig. S2). Absolute numbers for syncytial concentrations were obtained by fluorescence correlation spectroscopy (FCS) and showed a similar trend, deviating only at higher concentrations where FCS-based quantification is unreliable. We found that fusion-based dilution could be used to adjust cytoplasmic levels of an expressed protein over ~ two orders of magnitude. Importantly, larger dilutions brought cytosolic levels down to the sub-100 nM range, where the most quantitative and robust correlation spectroscopy measurements can be made.

**Figure 2:**
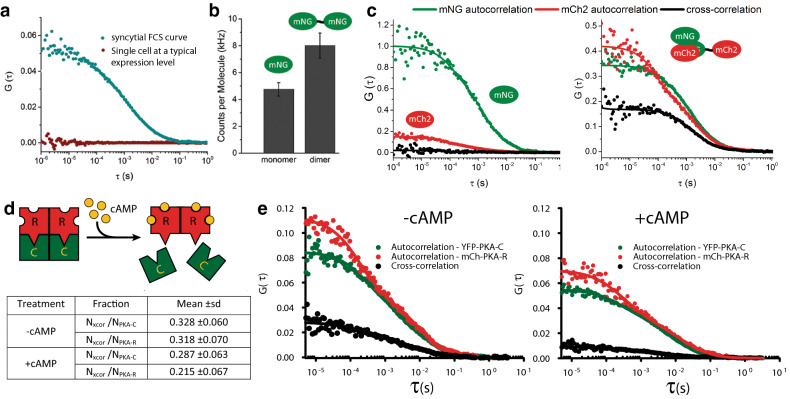
SPReAD improves in vivo fluorescence correlation spectroscopy (FCS) measurements. (**a**) FCS curves from U2OS syncytia are of uniform high quality since the concentrations can be set to FCS-compatible levels, compared to expression levels generally found in single transformed cells. (**b**) Brightness based on counts per molecule can be used to discriminate between monomeric and dimeric species in the cytoplasm of large syncytia and is useful for studying the stoichiometry of a single component within oligomers. (**c**) Cross-correlation spectroscopy is useful for studying heteromeric interactions. mNeonGreen and mCherry produce an appreciable cross-correlation (black line) when covalently joined (right) but not when co-transfected (left). In both cases, auto-correlations for each protein are clearly discernable. (**d**,**e**) FCCS in syncytia can be used to study functional differences in protein oligomerization. Protein Kinase A regulatory and catalytic subunits form complexes in the baseline state, repressing activity. Stimulation of adenylyl cyclase generates cAMP, causing PKA dissociation and increased activity. Data from syncytia created by mixing PKA-transfected U2OS cells with VSVG-U2OS cells. Table values in (**d**) represent data from 13 experiments.

To explore the benefits of using SPReAD for intracellular FCS measurements, we compared FCS data obtained from unfused cells with those from syncytia (Fig. 2a). In cells, transient mNG expression from a CMV promoter often fails to produce suitable autocorrelation curves, due to the high cytosolic concentration of labeled protein following transfection. FCS data is typically not usable when fluorophore levels exceed ~ 1 μM, which is well within the range of normal intracellular protein concentrations. In practice, one either picks cells with low enough expression to obtain usable correlation curves or carries out whole cell photobleaching to reduce the fluorescent species concentration to FCS-compatible levels^14^. Both of these options have clear biological drawbacks—either biasing the results by selecting only the low expressing cells, or phototoxicity from the bleaching method. We found that autocorrelations in unfused cells had an average dwell time of 2.2 ± 1.3 ms corresponding to a diffusion coefficient of 10 ± 5.8 μm^2^/s. We attribute the large deviations in the measured values (~ 50%) from overall poorer data quality due to the measurements being made at the higher than ideal fluorophore concentrations, and to altered mobility near bounding membranes or organelles within the single cells. We often saw artifacts due to photobleaching, which manifest as a change in G(0) over time (SI Appendix Fig. S4). In comparison, dwell times and G(0) values from syncytial data FCS curves showed much less variation due to the larger homogenous pool of diffusing fluorophores. Syncytial FCS curves yielded dwell times and diffusion coefficients (1.2 ± 0.1 ms; 13 ± 1.1 μm^2^/s) similar to the unfused cells but with much less variation.

Brightness analysis and two-color fluorescence cross-correlation spectroscopy are two valuable methods used to analyze protein–protein interactions within the cellular environment^12,15^. To evaluate these techniques in conjunction with cell fusion, we compared measurements made with covalent dimers of fluorescent proteins to the corresponding monomeric proteins. mNG dimers were found to be 1.7 times brighter than monomers (Fig. 2b). Assuming minimal quenching effects, this suggests a maturation efficiency of ~ 80% for mNG, which is on par with that of other green/yellow fluorescent proteins. Two-color fluorescence cross-correlation spectroscopy (FCCS) of mNeonGreen-mCherry2 covalent dimers yielded a 58% dimer population while a co-transfection of the monomeric proteins showed negligible cross-correlation amplitude (Fig. 2c). In addition to brightness and cross-correlation analyses, other methods such as photon counting histograms, dwell time distributions, photon anti-bunching and single-molecule FRET have been used to examine oligomerization states and could be aided by SPReAD sample preparation.

Next, we used syncytial two-color FCCS to study the oligomerization of protein kinase A (PKA), a Ser/Thr kinase that functions in the cAMP-dependent pathway of G-protein coupled receptor (GPCR) signaling. Upon GPCR activation, adenylyl cyclase catalyzes the conversion of ATP into cAMP, causing protein kinase A (PKA) regulatory subunits to dissociate from catalytic subunits, which are then free to phosphorylate downstream targets. Syncytial FCS of YFP-tagged catalytic subunits and mCherry-tagged regulatory subunits revealed a substantial cross-correlation, indicating functional repression in the baseline state (Fig. 2d,e). Upon stimulation with the adenylyl cyclase activator forskolin and the phosphodiesterase inhibitor IBMX, cross-correlation amplitudes decreased, reflecting cAMP-induced dissociation of subunits, and mirroring previous efforts using FCS in live cells or SiMPull with cell lysates^12,13,16^. SPReAD increases the usefulness and robustness of FCS and FCCS for cell-based measurements by allowing for target complex formation at more physiological intracellular concentrations and by mitigating complicating effects from confined cellular volumes.

### Single molecule imaging of membrane protein complexes

Most membrane proteins are freely mobile in two dimensions, unless tethered to intracellular actin. Protein complexes that reside in biological membranes are of significant interest to biomedical research, representing 23% of all ORFs in the human genome and being the target of > 60% of pharmaceutical drugs^17^. The biomedical significance of membrane receptors has motivated comprehensive investigation of their basic structures and mechanisms of action. Oligomerization is known to play a role in the function of many major receptor types (metabotropic, ionotropic and tyrosine kinases) and thus, considerable effort has been made to elucidate their interaction profiles. From a single-molecule perspective, subunit counting in oocytes has been the widely used approach, with many receptors being studied after controlled mRNA injection to limit receptor levels^18^. However, the concentration-dependence of oligomerization may be at odds with the sub-physiological expression levels employed in this technique and cell type specific post-translational modifications occurring in the Golgi and ER required for native oligomer formation may be lacking^19^. We show that cell fusion combined with single molecule imaging lifts this restriction and allows single molecule imaging after physiological assembly of receptor complexes in a cell type required by any specific biological constraints.

We undertook a series of experiments designed to demonstrate the unique utility of SPReAD for single molecule image-based measurements of membrane protein stoichiometry. In these experiments, we examined differences between the results obtained by SPReAD preparations and single molecule pulldowns to determine if detergent isolations had a notable effect, and also investigated the oligomeric state of several well-studied membrane protein complexes.

### Beta-2 adrenergic receptor stoichiometry

The adrenergic receptors (ADRβ1-3) are class A G protein coupled receptors (GPCRs) that are targets of catecholamines such as adrenaline and noradrenaline. Oligomerization of class A GPCRs is still controversial, with reports of ADRβ1 and ADRβ2 forming homodimers and heterodimers^20^; while more recent reports dispute this finding^21^. We used mNG-tagged ADRβ2 expressed in U2OS cells to assess dimer formation and compare our method to single molecule pulldown results. mNG-ADRβ2 expressing cells were co-plated with VSVG-expressing neighbors to dilute membrane receptors from the initial high expression levels. After cell fusion and incubation at 37 °C for 1-h, individual receptor complexes were clearly discernible and mobile within the plasma membrane, displaying similar kinetics to measurements made in living cells^22^ (Fig. 1b, SI Appendix Video 1). Single particle tracking of mNG-ADRβ2 (SI Appendix, Fig. S5) also confirmed this observation. The receptor concentration distribution was more heterogeneous across the syncytium than we saw with cytosolic proteins even ~ 1 h after fusion due to the slower diffusion rate for proteins in the membrane compared to cytoplasm. However, there were still numerous fields of view with uniform sparse distributions ideal for single molecule imaging (Fig. 1b, right panel).

**Figure 3:**
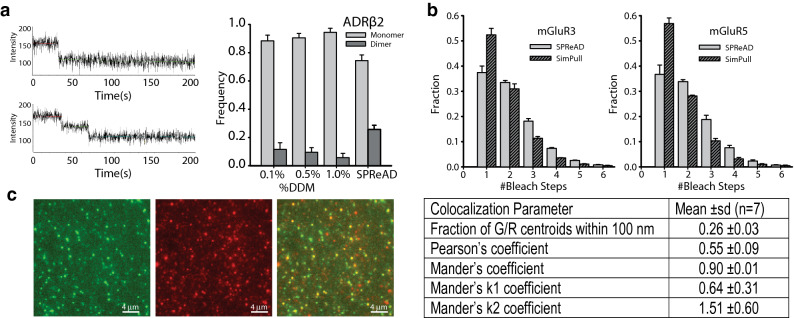
Single Protein Recovery After Dilution (SPReAD) for single molecule imaging avoids potential artifacts of detergent isolation. (**a**) Typical monomer (top-left) and dimer (bottom-left) mNG-ARβ2 bleach step traces obtained from the SPReAD-prepared samples. Right: The elimination of detergent isolation artifacts was demonstrated by measuring the mNG-ARβ2 dimer to monomer ratio from pull down experiments using mNG-ARβ2 isolated at three different detergent concentrations (first three bar groups) and from SPReAD preparations (fourth bar group). Data is the mean ± SEM, n = 3 experiments. A larger fraction of mNG-ARβ2 dimers were found using SPReAD, which we take to be a more accurate estimate of the physiological dimer ratio. (**b**) Comparison of metabotropic glutamate receptor (mGluR3 and mGluR5) complex stoichiometry in samples prepared via SPReAD, or detergent lysis (1% DDM) for SiMPull (mean ± SEM, n = 3 experiments, traces analyzed SPReAD: mGluR3 = 12,867; mGluR5 = 11,610; SiMPull: mGluR3 = 17,390; mGluR5 = 24,902). Similar to what we found with mNG-ARβ2, detergent-isolated single molecule pull-down experiments showed a larger monomer fraction relative to SPReAD. (**c**) Example of SPReAD-based two-color single molecule colocalization. Green, red and overlay images of mNG-ADRβ2 and mCherry-ADRβ1 revealing colocalization of adrenergic receptor subunits after cell fusion based on PSF colocalization analysis. Table: Averaged colocalization results from single molecule centroid based colocalization analysis and from conventional image-based colocalization methods (n = 7).

Syncytia were fixed with paraformaldehyde to immobilize receptor complexes and facilitate stoichiometry determination by stepwise photobleaching and two-color single molecule co-localization methods. mNG-ADRβ2 puncta showed distinct bleach steps (Fig. 3a, SI Appendix Fig. S6, top row). Analysis of the receptor population revealed that ADRβ2 was distributed between monomeric and dimeric states, with 25% of photobleaching traces showing two bleach steps (Fig. 3a, fourth bar group), signifying a 36% dimer population after accounting for mNG’s maturation efficiency (Supplementary Methods). In order to compare the effects of n-Dodecyl β-d-maltoside (DDM), a detergent commonly used in cell lysis for pulldown experiments we carried out single-molecule pull-down (SiMPull protocol) experiments on the same mNG-ADRβ2 expressing cells and found that dimer fractions differed from what was observed with SPReAD (Fig. 3a, first three bar groups). Using a standard detergent isolation and single molecule pulldown procedure the dimer fraction was never higher than 10%, indicating that in some cases detergent isolation methods can introduce significant errors in single molecule stoichiometry determinations.

### Heteromeric complex stoichiometry measurements of ADRβ2–ADRβ1 in fused cells

To demonstrate the ability to probe heteromeric associations with cell fusion and single molecule imaging, we co-expressed mNG-ADRβ2 and mCherry-ADRβ1 in U2OS cells. After fusion with VSVG-expressing cells both color channels showed distinct puncta in the syncytia and the overlaid image clearly displayed overlapping spots (Fig. 3c). The degree of colocalization was quantified by the standard pixel level methods (Pearson's and Mander’s coefficients) and at the single molecule level by PSF fitting and determining the fraction of spots with a nearest neighbor in the opposite color within 100 nm (Table in Fig. 3c). After fusion, the respective color channels showed a high degree of colocalization with 26% of mNG-ADRβ2 spots overlapping with mCherry-ADRβ2 spots as determined by centroid localization (Fig. 3c), which predicts a 40–50% colocalization, when corrected for missed colocalized pairs from non-fluorescent proteins. As reported by others^4^, the lower brightness and photostability of mCherry prohibited accurate stepwise photobleaching measurements in the red channel. However, based on the single molecule colocalization observed and statistical analysis of heterodimer formation we conclude that the affinity for ADRβ2–ADRβ1 heterodimer formation may be about equal to that of homodimer formation.

### Metabotropic glutamate receptor stoichiometry

The metabotropic glutamate receptors mGluR3 and mGluR5 are known to function as covalently bound homodimers via a cysteine bridge assembled in the ER prior to membrane trafficking^23^. We generated stable HEK293T cell lines constitutively expressing an mNeonGreen labeled metabotropic glutamate receptor, either mNG-mGluR3 or mNG-mGluR5, and co-plated them with VSVG-expressing U2OS cells. Fusion was induced and syncytia were formed and fixed with paraformaldehyde as described above. After fusion, individual receptor complexes were able to be resolved in many areas of the dish, demonstrating fusion between different cell types with proteins able to diffuse throughout the heterogeneous membrane of these syncytia.

Stepwise photobleaching experiments performed on the syncytia found that 33.5 ± 0.5% and 33.8 ± 0.8% for mNG-mGluR3 and mGluR5 respectively, suggesting that complexes formed prior to membrane trafficking were preserved during the fusion process (Fig. 3b), though these fractions are likely lower bounds on the actual dimer population. Single-molecule pull-down experiments were performed on the same cell lines using 1% DDM in the cell lysis buffer. In contrast to what we noted earlier for mNG-ADRβ2 when comparing isolation methods (Fig. 3a), we found less of a difference in the monomer-to-dimer ratio with both mGluRs. This may be due to disulfide linkages between mNG-mGluR monomers, which are known to be formed with endogenous mGluR5^23^. The mGluR data also showed a larger number of higher order oligomers than we saw with ADRβ2. We note that there is evidence for the formation higher order mGluR2 oligomers under certain conditions, as found using fluorescence fluctuation methods^24^.

Heterodimeric mGluRs have also been proposed and single molecule methods such as SPReAD would be a useful means of detecting them. Heterodimeric mGluRs would have important implications since the pharmacology of heterodimers may differ significantly from the better characterized mGluR homodimers^25^.

### Detection of higher-order oligomeric membrane protein complexes: CRAC channel subunit Orai1 stoichiometry

**Figure 4:**
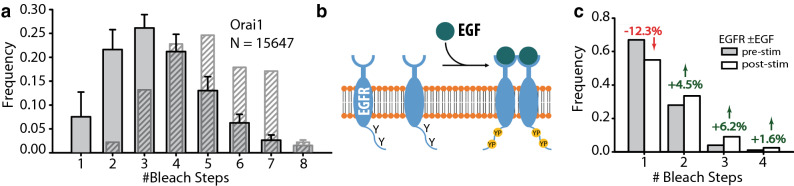
Application of SPReAD to detect higher-order oligomer membrane protein complexes and measure stoichiometric changes due to ligand binding. (**a**) The functional form of the CRAC channel continues to be a contested issue, with claims that Orai1 subunits adopt either a tetrameric or a hexameric configuration. The raw bleach step data from 14,888 fluorescent spots analyzed from SPReAD syncytia made from Orai1 expressing cells yielded a weighted mean step number of 3.5 (gray bars). Hatched bars represent an estimate of the actual subunit fractions assuming a 0.8 fluorescent fraction for mNG and indicate a weighted mean subunit number of 5.0. (**b**) Epidermal growth factor binding stimulates EGFR dimerization and autophosphorylation of tyrosine residues on EGFR’s cytoplasmic tail, leading to recruitment of downstream signaling proteins. (**c**) SPReAD bleach step histograms of EGFR oligomerization before (gray bars) and after (white bars) EGF stimulation. Although EGFR is largely monomeric prior to growth factor addition, there is a substantial dimer fraction as well. After stimulation, the dimer and higher-order oligomers fractions increase, while the monomer fraction drops.

We also examined the subunit stoichiometry of Orai1, a calcium-selective ion channel that forms the central pore of the calcium release-activated channel. The functional stoichiometry is currently unresolved, with claims of either a tetrameric or a hexameric configuration^26,27^, but in either case, it is an example of a protein complex that is a higher order oligomer. Using SPReAD we found that most Orai1 puncta bleached in 1–6 steps (Fig. 4a). Assuming an 80% successful protein folding rate (Supplementary Methods), the corrected distribution (diagonal marked bars in Fig. 4a) has a weighted mean of five Orai1 subunits per complex. Although the average number of subunits found lies between 4 and 6, we take this as the lower bound of the oligomer order. Single molecule protein stoichiometry determinations can be affected by factors such as steric interference by the fluorescent protein moiety and/or the presence of endogenous protein of interest. Targeted knockdown of endogenous proteins, careful choice of cell lines, or fluorescent labeling of endogenous Orai1 may be used to refine the understanding of physiologically relevant oligomerization in specific tissue types.

### Ligand-dependent oligomerization of epidermal growth factor receptor

One of the primary evolutionary advantages conferred by oligomerization is the development of new modes of regulatory control of protein activity. Allosteric oligomerization is known to play a role in the mechanisms of both metabotropic receptors and receptor tyrosine kinases, with extracellular ligands modulating the formation of dimers or higher order structures. Monomer-oligomer transitions can prime receptors for downstream signaling events, such as post-translational modifications or the recruitment of adaptor proteins. As an example of the use of SPReAD to detect ligand-dependent multimerization, we looked at epidermal growth factor receptor—a member of the receptor tyrosine kinase family whose abnormal regulation has been implicated in a number of human cancers^28^. The canonical model for receptor activation asserts that EGFR is monomeric in the plasma membrane prior to stimulation, whereupon it is driven to dimerize upon binding of its cognate ligands, resulting in autophosphorylation of tyrosine residues on its cytoplasmic tail and recruitment of specific effector proteins (Fig. 4b). Although the EGFR pathway has been extensively studied using both bulk and single molecule approaches, there are still open questions about receptor oligomerization. There is increasing evidence that pre-formed dimers of EGFR exist on the cell surface prior to ligand stimulation and that EGFR is capable of forming higher-order oligomers that may function in receptor activation^29–31^. To examine each of these possibilities, we expressed a mNeonGreen-tagged EGFR (mNG-EGFR) on the cell surface and performed stepwise photobleaching measurements in fixed syncytia. Even in its baseline state, EGFR was found to be significantly dimeric, with 29% of traces bleaching in two steps (Fig. 4c), though it is possible that weakly interacting subunits may dissociate after dilution, making this a lower bound on oligomerization. Upon stimulation with EGF, this dimer fraction increased and higher-order oligomers (trimers and tetramers) were also observed. Together, these results support a model where at least some EGFR signaling is accomplished by conformational changes in pre-formed dimers and trans-activation by higher-order oligomers. The use of SPReAD to study ligand-dependent oligomerization of EGFR validates its potential for studying transient interactions.

## Discussion

By achieving detergent-free dilution of protein complexes after physiological assembly, SPReAD facilitates measurements of subunit stoichiometry for both cytosolic and membrane-bound oligomers. Furthermore, the use of VSVG as a means of accomplishing cell fusion is highly efficient and requires only a simple buffer exchange. In contrast, existing methods for probing oligomerization are significantly more complex or disruptive. Use of stimulated emission depletion to reduce excitation volumes by > 100-fold can extend the upper limit on FCS measurements^32^ but requires complicated optics and increases photobleaching and phototoxicity. As mentioned earlier, single molecule pull-down approaches can probe molecular heterogeneity in oligomerization but require extraction of protein complexes from their native environment^4,33^. Meanwhile, efforts to apply conventional imaging or localization microscopy to stoichiometry analysis rely on a priori assumptions about protein distribution or fluorophore blinking^34,35^. Compared against these other methods, SPReAD offers unique advantages, affording single molecule sensitivity for oligomerization studies while maintaining a more physiologically relevant setting.

Although the use of cell fusion for dilution is both simple and rapid, the dynamics of various intracellular processes need to be considered when interpreting results. Syncytia form almost instantly after pH drop, but protein redistribution is diffusion-limited and thus, much slower for membrane-bound proteins undergoing 2D diffusion compared to cytosolic proteins moving much more rapidly in 3D. This yields two possible modes of analysis: an equilibrium mode, where the final concentration of labeled protein complexes is uniform and proportional to the initial concentration (divided by the co-plating ratio), and a non-equilibrium mode, where concentrations across the imaging dish are non-uniform. The latter mode, typically carried out 10–45 min after buffer exchange for the proteins studied here, is most conducive to measuring subunit stoichiometry as it minimizes time during which non-covalent protein complexes can dissociate. However, since this time is nonzero, it is important to acknowledge that weakly interacting subunits may dissociate between the time of cell fusion and stoichiometry measurements, signifying that our data likely represent lower bounds on protein oligomerization. Beyond these considerations of diffusion and dissociation, syncytia appeared to be morphologically stable for 5–6 h but it is still largely unclear how the intracellular environment is reshaped during the fusion process. Understanding how syncytium formation affects major signaling pathways will be critical for proper interpretation of SPReAD results.

The plasma membrane is a unique and crowded environment composed of lipids, proteins, and other biomolecules. This is further complexified by the existence of lipid rafts, ordered and disordered regions, and regions with contact to cytoskeletal elements. This environment is reshaped during the fusion process, and therefore there may be an impact on the structure and function of some protein complexes if two different cell types are being fused so that resulting membrane composition of the syncytia formed will be a mixture of the two parent cells. For example, in some cases the binding of specific lipids to large, polytopic membrane proteins can be crucial for protein complex stability^36^ and the mixing of lipid types between cells could skew the oligomerization state. We note that in this work we have carried out fusions using the same cell type (U2OS) except the mGluR experiments where HEK293 cells where used to express the mGluR complexes.

Another concern with our approach may be that the use of a brief pH drop to initiate VSVG-mediated cell fusion may affect complex stoichiometry. We note that in our comparisons between SPReAD and single molecule pulldowns, we consistently saw higher levels of oligomerization using our cell fusion method compared to detergent isolated preparations, indicating that the pH jump is at least less disruptive than detergent pulldown for the proteins we studied. Furthermore, despite the extracellular pH drop, we found that intracellular pH is largely unchanged (SI Appendix Fig. 8), and therefore proteins and protein complexes en route to the membrane are likely unaffected. Proteins trafficked to the membrane during the fusion time course occurring after the pH jump would be able to associate normally for complexes that oligomerize on the membrane (e.g. EGFR). It should also be possible to achieve in situ protein dilutions similar to what we show here using alternative viral fusogens, such as Reovirus Fast proteins^37^, which do not require a pH jump to activate.

A number of strategies may be used to augment the SPReAD technique and build upon its versatility. In the experiments discussed here, we over-expressed fluorescently tagged versions of the proteins of interest in order to measure subunit stoichiometry; however, over-expression and the presence of endogenous unlabeled subunits can bias the results. For more accurate measurement of physiologically relevant interactions, endogenous proteins can be directly labeled by genome editing or primary cells can be extracted from genetically modified organisms to understand tissue-specific phenotypic variation. Future work may also extend SPReAD applications beyond the cytoplasm and plasma membrane by making use of membrane contact sites between organelles, examining proteins that exchange between the cytoplasm and other compartments or by retargeting of proteins through signal sequence engineering.

By removing limits on expression levels compatible with single molecule experiments without requiring chemical agents for dilution, SPReAD permits minimally perturbative measurements in a variety of cell lines. Aside from the FCS- and stepwise photobleaching-based analyses of subunit stoichiometry highlighted here, we expect SPReAD to enhance other methods traditionally limited to working at low concentrations such as smFRET, single-particle tracking and single molecule spectroscopy, thus providing a powerful addition to the single molecule toolkit.

## Methods

### Cloning of inducible VSVG and labelled proteins

To avoid the deleterious effects of long-term VSVG expression, the coding sequence for VSVG (Addgene #8454) was cloned into the BamHI and EcoRI sites of the lentiviral pLV Puro Tet vector for doxycycline-inducible expression. A constitutively expressed mNeonGreen lentiviral plasmid was produced by excising mNeonGreen from mNeonGreen-N1 (Allele Biotech) using NheI and NotI and subcloning into pCDH-puro (System Biosciences). This resulting plasmid has been deposited with Addgene (plasmid #82724).

Synthetic dimers of fluorescent proteins were produced by placing a helical linker A(EAAAK)_5_A after the mNG sequence in mNG-C1 (between the BspEI and BglII sites). mNG or mCh2 were then PCR amplified and placed after this linker (between NotI and SpeI sites) to generate mNG–mNG or mNG–mCh2, respectively. The rigid helical linker spaces the fluorescent protein domains further apart to reduce energy transfer^38,39^. pCDH-puro and mNG-C1 were both digested with NheI and BamHI to excise the fluorescent protein and place it into the pCDH lentiviral plasmid to generate pCDH-puro-mNG, which was used to produce a stable mNG cell line.

mNG-tagged ADRβ2 and EGFR were generated by cloning into the pSNAPf-ADRβ2 backbone (New England Biolabs). mNG was amplified by PCR from mNG-C1 and placed between the EcoRI and SbfI sites of pSNAPf-ADRβ2 (replacing the SNAP tag) to yield mNG-ADRβ2. Site-directed mutagenesis was used to remove a ClaI site from wildtype EGFR. This mutated EGFR was then PCR amplified and placed between the SbfI and XhoI sites of the pSNAPf-ADRβ2 plasmid, replacing ADRβ2. The EGFR signal sequence was purchased as a gBlock (Integrated DNA Technologies) and placed between the ClaI and BmtI sites to generate mNG-EGFR. Lentiviral versions of mNG-ADRβ2, mNG-EGFR, mNG-mGluR3, and mNG-mGluR5 were produced by amplifying each plasmid by PCR and digesting with XbaI and NotI to place the fusion protein after the CMV promoter in pCDH-puro. To make Orai1-mNG, Orai1-YFP (Addgene #19756) and mNG-N1 were digested with AgeI and NotI to remove YFP and replace it with mNG.

### Cell culture and generation of stable cell lines

U2OS human osteosarcoma cells were cultured in DMEM without phenol red, supplemented with 10% fetal bovine serum (FBS), sodium pyruvate, 1 × GlutaMax and 1 × antibiotic–antimycotic; all cell culture media and supplements were purchased from Life Technologies. For stable expression of VSVG under tetracycline control, U2OS cells were first stably transduced with the rtTA NeoR plasmid for the reverse tetracycline-controlled transactivator (rtTA) protein. Lentiviral particles were generated in HEK293 cells and used to transduce U2OS cells as previously described^32^. Stably transduced cells were selected using 700 μg/mL G418. U2OS rtTA cells were then transduced with pLV puro Tet-VSVG and selected using 2 μg/mL puromycin. Doxycycline was withheld from cell culture media until 24 h prior to cell fusion. Stable mNeonGreen cell lines were produced by transducing U2OS Tet-VSVG cells with pCDH-puro-mNG-C1, pCDH-puro-mNG-ADRβ2 and pCDH-puro-mNG-EGFR and selecting with 2 μg/mL puromycin. Stable mNeonGreen-mGluR expressing HEK293T cell lines were produced by transducing cells with either pCDH-puro-mNG-mGluR3 or pCDH-puro-mNG-mGluR5 and enriched by fluorescence-activated cell sorting (BD Biosciences FACSAria).

### Substrate preparation

To minimize glass autofluorescence and maximize cell attachment, plain glass-bottom dishes were cleaned and coated with fibronectin. Dishes were etched with 1 M KOH for 20 min, followed by DIO water and then PBS rinse. For fibronectin coating, dishes were incubated in 4% (3-Mercaptopropyl)trimethoxysilane (Sigma-Aldrich) in ethanol for 30 min, rinsed with ethanol, incubated with (*N*-γ-maleimidobutyryl-oxysuccinimide ester) crosslinker (4 mM in ethanol, Thermo Scientific), rinsed with ethanol and dried thoroughly in a sterile biosafety cabinet. Dishes were then incubated with 5 μg/mL fibronectin for 2 h at room temperature, followed by overnight at 4 °C, then rinsed with PBS and stored in PBS at 4 °C until use (up to several weeks).

### Fusion assay

U2OS Tet-VSVG cells were plated onto collagen coated glass-bottom dishes. After reaching confluence, fresh media with 2 μg/mL doxycycline was added and the cells were returned to a CO_2_ incubator for 24 h. Cells were then fused by removing culture media, washing with PBS and incubating in fusion buffer (PBS with 25 mM MES, pH 5.5) for 1 to 5 min. Cells were washed with PBS and culture media was restored before returning cells to the CO_2_ incubator. Cell membranes and nuclei were labelled at various time points by incubating with 5 μg/mL Wheat Germ Agglutinin Alexa 647 (Life Technologies) and 5 μg/mL Hoechst 33,342 in Hank’s balanced salt solution for 10 min prior to fixation with 4% paraformaldehyde. Fixed cells were imaged on a spinning disk confocal microscope (Olympus) with air objectives (40 ×/0.9, 20 ×/0.7 and 10 ×/0.4) and examined for syncytia formation.

### Confocal microscopy and fluorescence correlation spectroscopy

U2OS Tet-VSVG cells were transfected with FP control plasmids or FP-tagged PKA-subunits using Lipofectamine 3,000; for cytoplasmic mNG measurements, stable U2OS mNG cells were used to accurately control the number of expressing cells. Serum-free Fluorobrite DMEM (Life Technologies) was used to minimize cellular autofluorescence. The two were mixed at various ratios and 5 × 10^5^ cells were plated in the well of a 14 mm diameter glass-bottom dish (collagen/fibronectin-coated) using doxycycline-supplemented media (2 μg/mL); additional media was added 2–12 h after plating, after cells were visibly attached and spread. Cells were imaged on a confocal microscope (Zeiss LSM880). Fluorescence correlation spectroscopy was performed on the same instrument using the LSM880 32-channel GaAsP detector in photon counting mode. Standard FCS fitting equations were used^12^ and further details of FCS data analysis are detailed in the SI Appendix. For non-PKA FCS measurements, the data was fit to a single component diffusion with a triplet model. Absolute concentrations for cytoplasmic mNeonGreen were obtained by calibrating the focal volume with known concentrations of Alexa 488. From the two-color cross-correlation measurements, the average number of particles was determined using:

$$N_{G,R} = \frac{1}{{G\left( 0 \right)_{G,R} }} \quad \frac{{N_{x} }}{{N_{G} }} = \frac{{G\left( 0 \right)_{x} }}{{G\left( 0 \right)_{R} }} \quad \frac{{N_{x} }}{{N_{R} }} = \frac{{G\left( 0 \right)_{x} }}{{G\left( 0 \right)_{G} }}$$

where N_G,R_ is the number of green or red particles, and N_X_/N_G_ and N_X_/N_R_ are the heterodimer fractions. For Protein Kinase A experiments, PKA-transfected U2OS cells were mixed 1:10 with non-expressing VSVG cells and incubated in doxycycline-supplemented Fluorobrite DMEM for 24 h. Cells were then fused by a 5-min incubation in fusion buffer and FCS was performed in syncytia 1 h later. In order to maintain the same syncytial position for post-stimulation measurements, 2 × cAMP-stim buffer (50 μM forskolin, 200 μM IBMX in Fluorobrite DMEM) was added directly to the imaging dish in equal volume to the residual media and a second FCS recording was initiated 5 min later. PKA data was fit to a two-component diffusion model (10).

### Single-molecule imaging after cell fusion

U2OS Tet-VSVG cells were transfected with FP-tagged receptor constructs and plated onto glass-bottom dishes with non-transfected cells at a ratio of 10:1 (non-transfected:transfected), as described above. After 24 h of doxycycline induction, cells were fused and imaged live (1–2 h after fusion) or fixed for stoichiometry/colocalization analysis. Cells were fixed with 4% paraformaldehyde for 3 h in the dark at room temperature to eliminate residual mobility of membrane proteins after short fixation^33^. For mNG-EGFR experiments, the syncytia were stimulated with 200 ng/mL EGF 75 min after cell fusion was initiated and fixed 5 min later or fixed without EGF treatment.

### TIRF Microscopy

A custom-built azimuthal-scanning objective-TIRF microscope was used for single molecule imaging. Excitation at 488 nm and 561 nm were used to excite mNeonGreen and mCherry, respectively, and were directed to the sample using a quad polychroic (ZT405/488/561/640rpc, Chroma Technology) housed in the filter wheel. A beam telescope and focusing lens were used to create a collimated beam out of the objective (Olympus UApoN 100x/1.49), while a pair of XY galvanometer mirrors (Model 3210H, Cambridge Technology) controlled the angle of incidence. For the detection path, a TuCam adaptor (Andor) equipped with band pass filters (ET525/50 for mNeonGreen and ET605/52 m for tdTomato) was used to split emissions onto two EMCCDs (Andor iXon 887 and 897 Ultra). Image coregistration was accomplished by acquiring brightfield images of a calibration objective (Zeiss LSM calibration objective) prior to each imaging experiment and ensuring that the images were coregistered to better than one pixel over the camera field-of-view through alignment of the detection pathway. Live-cell data was acquired at 37 °C using an objective heater (Bioptechs), while fixed cell experiments were performed at room temperature. Coverslips were scanned for regions with a suitable density of molecules for single molecule analysis; regions with unfused fluorescent cells or too few/too many molecules were avoided. For bleach step analysis, 2000 frames were recorded at 10–30 Hz; laser intensity was kept low to mitigate blinking artifacts. For colocalization analysis, 20 frames were acquired and averaged during post-processing.

### Single molecule data analysis

Photobleaching movies were analyzed using three different methods. Two are based on the use of a custom lab software package (ImageC.exe, written in C/C +  + under Microsoft Visual Studio 2017) and the third was the use of a published software called Progressive Image Filtering (PIF) which is an automated software package written in Matlab^40^. In both programs, molecules (PSFs) were first located automatically found by successive processing of the summed image stack to locate fluorescent puncta above a certain threshold that meets a specified Gaussian fit criterion. For each molecule, an ROI (typically 5 × 5) centered on the pixel containing the PSF centroid was created and the ROI mean values vs. time (frame) extracted from the stack. The ROI center pixel coordinate was readjusted slightly as needed as the data is extracted from the frames so that the brightest pixel is always at the center. ROI fluorescence traces of all the spots located are stored within the program and displayed as time trace plots for (1) manual (i.e. “by-eye”) step counting in ImageC, or used with the automated step-finding algorithms in (2) ImageC or in (3) the PIF software package obtained from the Blunck lab at Université de Montréal. Both algorithms count the number of bleach steps based on signal noise and a user-set change in the trace count level that determines a valid step. Traces without discernible bleach steps were discarded. At least 700 molecules were analyzed for each sample, with > 10,000 traces typically analyzed when using the two automated software’s. We consider the by-eye approach to be the “gold standard”, and use it to initially select algorithm parameters that produce distributions that match the human scored data. We compared results obtained on the same data set using all three methods and found that all returned the same distributions with only no significant differences. Further information on the programs used is provided in the Supplementary Methods section and in SI Appendix Fig S7.

For colocalization analysis, data from two EMCCDs were analyzed to find spots in both the green and red channels using either a custom MATLAB script or function built into our lab’s custom analysis program (ImageC). The PSFs were fit to a Gaussian model to determine center locations. A colocalization fraction was calculated to be the fraction of mNeonGreen spots with an mCherry spot less than 100 nm away.

## Supplementary information

Supplementary Information.

Supplementary Video 1.

Supplementary Video 2.

Supplementary Video 3.
