# Supplementary material for: Stoichiometric analysis of protein complexes by cell fusion and single molecule imaging
Source: Sci Rep. 2020 Sep 10;10:14866. doi: 10.1038/s41598-020-71630-6 (PMC7483473; doi:10.1038/s41598-020-71630-6)
Supplement: Supplementary file 1 — Supplementary Information. [file 41598_2020_71630_MOESM1_ESM.pdf]

Title: Stoichiometric Analysis of Protein Complexes by Cell Fusion and Single Molecule Imaging

Authors: Avtar Singh[a,†], Alexander L. Van Slyke[b,‡], Maria Sirenko[c,§], Alexander Song[a¶], Paul J. Kammermeier[d], Warren R. Zipfel[a,b,e,*]

[a]Applied and Engineering Physics, Cornell University, Ithaca, NY 14853
[b]Graduate Field of Biophysics, Cornell University, Ithaca, NY 14853
[c]Department of Biological Engineering, Cornell University, Ithaca, NY 14853
[d]Department of Pharmacology and Physiology, University of Rochester Medical Center, Rochester, NY
[e]Meinig School of Biomedical Engineering, Cornell University, Ithaca, NY 14853

[†]Current Address: Broad Institute, Cambridge, MA 02142
[‡]Current Address: University of Pennsylvania, Philadelphia, PA 19104
[§]Current Address: Memorial Sloan Kettering Cancer Center, New York, NY 10065
[¶]Current Address: Max Planck Institute for Intelligent Systems, Stuttgart, Germany

SUPPLEMENTAL METHODS

Time course imaging of cell fusion dynamics.

The kinetics of cell fusion and subsequent protein diffusion were thoroughly investigated to guide the timing of single molecule experiments. These dynamics will vary for each protein of interest and thus, similar experiments may need to be conducted prior to FCS or stepwise photobleaching measurements. As an example of cytoplasmic proteins, U2OS cells expressing mNG were co-plated with non-expressing VSVG cells at a ratio of 1:10 or 1:100. Cells were fused using a 30-second incubation in fusion buffer and then returned to imaging media (Fluorobrite DMEM). The imaging dish was loaded onto a confocal microscope (Zeiss LSM 880) and a time-series was started 2 minutes after fusion was initiated. Both the 1:10 and 1:100 co-plating ratios were imaged at room temperature (23°C), while the former was also imaged at 37°C to examine the effects of temperature on protein diffusion.

Diffusion of mNG-ADRβ2 was investigated by fixing syncytia at various time points after cell fusion. Fusion was accomplished by a 30-second incubation in fusion buffer and cells were then returned to imaging media (Fluorobrite DMEM) at 37°C. Cells were fixed with 4% PFA for 3 hours, then washed with PBS, and imaged on a commercial TIRF microscope (Zeiss Elyra).

A custom-built brightfield microscope housed in a CO_2 incubator was used to examine the time-course of cell fusion. The microscope consists of an x-y stage (Applied Scientific Instrumentation), ring illuminator and camera (Chameleon, Point Grey Research). Custom software allows for autofocus and tiling of the culture dish.

U2OS Tet-VSVG cells were plated and induced with doxycycline for 24h as described earlier. Cells were fused by a brief 30-second incubation in fusion buffer, washed with PBS and restored to normal culture media. Immediately, the dish was loaded into the incubator microscope and imaged for ~30 hours.

FCS/FCCS data analysis. For non-PKA experiments, data was fit to a single component diffusion with triplet model:

$$G(\tau) = G(0) \cdot \left(1 - F_T + F_T\, e^{-\frac{\tau}{\tau_T}}\right) \cdot \left[\left(\frac{1}{1 + \frac{\tau}{\tau_d}}\right) \cdot \sqrt{\frac{1}{1 + \frac{\tau}{S \cdot \tau_d}}}\right]$$

where τ_T and F_T are the triplet time and fraction, respectively, τ_d is the diffusion time, S is the structure factor for the focal volume and G(0) is the correlation at $\tau = 0$. The structure factor was set to 10 for all fits. From the two-color cross-correlation measurements, the average number of particles was determined using:

$$N_{G,R} = \frac{1}{G(0)_{G,R}} \qquad \frac{N_x}{N_G} = \frac{G(0)_x}{G(0)_R} \qquad \frac{N_x}{N_R} = \frac{G(0)_x}{G(0)_G}$$

where $N_{G,R}$ is the number of green or red particles, and N_X/N_G and N_X/N_R are the heterodimer fractions. Absolute concentrations for cytoplasmic mNeonGreen were obtained by calibrating the focal volume with known concentrations of Alexa488.

For Protein Kinase A experiments, PKA-transfected U2OS cells were mixed 1:10 with non-expressing VSVG cells and incubated in doxycycline-supplemented Fluorobrite DMEM for 24 hours. Cells were then fused by a 5-minute incubation in fusion buffer and FCS was performed in syncytia one hour later. In order to maintain the same syncytial position for post-stimulation measurements, 2x cAMP-stim buffer (50 μM forskolin, 200 μM IBMX in Fluorobrite DMEM) was added directly to the imaging dish in equal volume to the residual media and a second FCS recording was initiated 5 minutes later. PKA data was fit to a two-component diffusion model:

$$G(\tau) = G(0) \cdot \left\{\left[\left(\frac{\alpha}{1 + \frac{\tau}{\tau_{d1}}}\right) \cdot \sqrt{\frac{1}{1 + \frac{\tau}{S \cdot \tau_{d1}}}}\right] + \left[\left(\frac{1-\alpha}{1 + \frac{\tau}{\tau_{d2}}}\right) \cdot \sqrt{\frac{1}{1 + \frac{\tau}{S \cdot \tau_{d2}}}}\right]\right\}$$

where τ_{di} are the diffusion times for the respective fractions (i = 1, 2), α is the fractional contribution of the first component and all other parameters are as defined above.

Single particle tracking of membrane proteins in giant syncytia. Membrane proteins are typically free to diffuse in the plasma membrane unless tethered to larger intracellular structures. To examine protein mobility in giant syncytia, U2OS cells expressing mNG-ADRβ2 were co-plated with non-expressing VSVG cells and immersed in fusion buffer for 5 minutes before returning them to Fluorobrite DMEM and incubating at 37°C for 75 minutes. Cells were imaged at 30 fps on a lab-built TIRF microscope at 37°C with an EMCCD (iXon 887, Andor).

Single molecule trajectories were analyzed using the ImarisTrack module in Imaris Bitplane. The raw data was temporally averaged by five frames to improve signal-to-noise, yielding a substack interval of 150 ms, and SPT parameters (PSF quality and size) were chosen based on the dataset. Spots were detected in each substack frame and trajectories were assembled with an autoregressive motion model. 4580 trajectories lasting longer than five substack frames were generated and included for further analysis. MSD plots were fit to extract single particle diffusion coefficients (MSD = 4Dt), which were distributed exponentially. Trajectories were sorted by their

diffusion coefficient, heuristically classified into slow, intermediate and fast motions (bottom, middle and top thirds) and average MSD plots were calculated for each of these fractions.

mNeonGreen folding efficiency and true oligomer calculations. Our FCS data of mNeonGreen (mNG) monomers and covalent dimers permitted an estimation of the fluorescent protein maturation efficiency, denoted as f. In the case of monomers, misfolded proteins are not detected and thus, the detected brightness-per-particle is equal to the brightness of the monomeric species. For simplicity, we normalize this to 1.

$$B_1 = 1$$

In the case of dimers, we observe a mixture of monomers and dimers. The overall brightness-per-particle (count rate divided by average number of particles) is given by:

$$B_{mix} = 2f(1-f)B_1 + f^2 B_2$$

where the prefix to each term denotes the probability of an mNG dimer having one or two fully mature fluorescent domains. Assuming $B_2 = 2B_1 = 2$, this simplifies to:

$$B_{mix} = 2f(1-f) + 2f^2 = 2f$$

Using our normalized value for B_{mix} (1.7, the observed count rate for dimers divided by count rate for monomers), we estimate that mNeonGreen folds to 80 - 85% efficiency.

We can use this fraction to estimate the true dimer propensity of ADRβ2, which was largely present in monomeric and dimeric forms. If we denote the true monomer and dimer fractions as χ_1 and χ_2, then we obtain the relation

$$\chi_1 + \chi_2 = 1$$

assuming no higher-order oligomers exist.

The "emitter fractions" χ_i' are given by considering that each ADRβ2 oligomer can have properly folded or misfolded mNG domains. The fractions are given by:

$$\chi_0' = (1-f)\chi_1 + (1-f)^2\chi_2$$

$$\chi_1' = f\chi_1 + 2f(1-f)\chi_2$$

$$\chi_2' = f^2\chi_2$$

It is important to note that these emitter fractions are not the directly observed quantity (largely because χ_0' cannot be detected). Instead, we detect

$$\phi = \frac{\chi_1'}{\chi_1' + \chi_2'}$$

For ADRβ2, $\varphi = 0.75$. From the above system of linear equations, we can uniquely determine the values of the "true" monomer and dimer fractions, χ_1 and χ_2 (0.64 and 0.36 for ADRβ2).

Generalized Dark Fraction Correction. Assuming a constant fluorescent protein dark fraction independent of oligomerization state, the observed fractions of each state can be corrected for the presence of dark protein components as an estimate of the actual fraction.

p = fraction of fluorescent proteins (0 to 1.0)

1 - p = dark fraction

The fractional losses of each n-mer state are given by binomial coefficients. These form a series of linear equations that are solved to correct the observed fractions. The matrix form, written here for a maximum oligomer state of 6 (k):

$$\begin{bmatrix} U(1,1) & U(1,2) & U(1,3) & U(1,4) & U(1,5) & U(1,6) \\ 0 & U(2,2) & U(2,3) & U(2,4) & U(2,5) & U(2,6) \\ 0 & 0 & U(3,3) & U(3,4) & U(3,5) & U(3,6) \\ 0 & 0 & 0 & U(4,4) & U(4,5) & U(4,6) \\ 0 & 0 & 0 & 0 & U(5,5) & U(5,6) \\ 0 & 0 & 0 & 0 & 0 & U(6,6) \end{bmatrix} \begin{bmatrix} a_1 \\ a_2 \\ a_3 \\ a_4 \\ a_5 \\ a_6 \end{bmatrix} = \begin{bmatrix} b_1 \\ b_2 \\ b_3 \\ b_4 \\ b_5 \\ b_6 \end{bmatrix}$$

Where the upper triangular matrix (**U**) elements are:

$$U(n,k) = \frac{n!}{r!(n-k)!} p^k (1-p)^{n-k} \quad n \equiv \text{number of subunits} \quad k \equiv \text{ subunit number (1 to max assumed)}$$

The vector *b* is the observed number of steps (oligomer subunits) and the vector *a* is the corrected number of subunits per complex. ***Ua = b*** can be solved for ***a*** by back-substitution since all of the diagonal elements are non-zero.

Analysis software for bleach step quantification.

ImageC: ImageC.exe is a lab-written image analysis program that has single molecule/centroid localization functions useful for single molecule analysis. The application is a Windows based program written in C/C++ using Microsoft Visual Studio 2017. For a portion of the bleach step analysis used in this work, ImageC was used to two modes: (1) user-determined number of steps based on observation of time traces, where the users tally the results within a program spreadsheet, or (2) an automated bleach step-counting algorithm that is described in Figure S7.

Spot location method. Single molecule spots are located by analysis of an image created by summation of a subset of the first 20-50% of image frames. A histogram of the summed image is calculated and pixels at the maximum pixel value located, analyzed either on a peak vs background levels basis for the NxN box (typically 5x5 pixels), or using a Gaussian mask-based merit function described below. Once the bright center pixel is analyzed, the pixels within the NxN box are set to zero. The level criteria determine whether the peak pixel is greater than the user-set background value (BG) for all the pixels within the NxN box. The Gaussian difference mask

method compares the NxN pixels surrounding the peak pixel to see if they conform to a Gaussian profile relative to the center of the spot. The extent of conformance is a user-selectable value that is the fractional ±amount the pixel can deviate from the expected value, based on what would be expected if the spot were a perfect Gaussian. If a spot has nearest neighbor pixels that do not meet this criterion, the spot is not used.

Gaussian Difference Mask (GDM) method. Assume a Gaussian PSF with a 1/e radius of σ centered at pixel i = j = 0. The camera pixel size is p, and p and σ are in the same units. The pixels at and near the center of the PSF would have values of:

$$\mathrm{Pixel}(i,j) = B_{i,j} + \left(A_{0,0} - B_{i,j}\right)\frac{\left[\left(\mathrm{erf}\left(ip+\frac{p}{2}\right) - erf\left(ip-\frac{p}{2}\right)\right)\left(\mathrm{erf}\left(jp+\frac{p}{2}\right) - erf\left(jp-\frac{p}{2}\right)\right)\right]}{4\,\mathrm{erf}\left(\frac{p}{2\sigma}\right)^2}$$

The above can be used to pre-calculate a mask for a given σ and pixel size, which can then be applied as a merit function to judge whether a spot on an image is a PSF:

$$\mathrm{Mask}(i,j) = \frac{\left[\left(\mathrm{erf}\left(ip+\frac{p}{2}\right) - erf\left(ip-\frac{p}{2}\right)\right)\left(\mathrm{erf}\left(jp+\frac{p}{2}\right) - erf\left(jp-\frac{p}{2}\right)\right)\right]}{4\,\mathrm{erf}\left(\frac{p}{2\sigma}\right)^2} \quad \text{Merit func.} \equiv \sum_{0}^{N}\left(P_{i,j} - B + \left(A_{0,0} - B\right)Mask(i,j)\right)^2$$

PIF[40]: In brief, spots are located by scanning a Laplacian of a Gaussian kernel across the surface. Fluorescent puncta above a certain threshold with a good Gaussian fit are selected as valid spots for analysis. The intensity vs time traces of these spots are analyzed using an algorithm which locates intensity drops larger than a predetermined threshold in the trace. A level is determined as the average value of the trace between two drops. The intensity difference between adjacent levels is then compared and they are combined if it is below the threshold. This is repeated in an iterative manner until all remaining levels are separated by an amount greater than the threshold. Each image contained ~600 spots.

SUPPLEMENTAL FIGURES

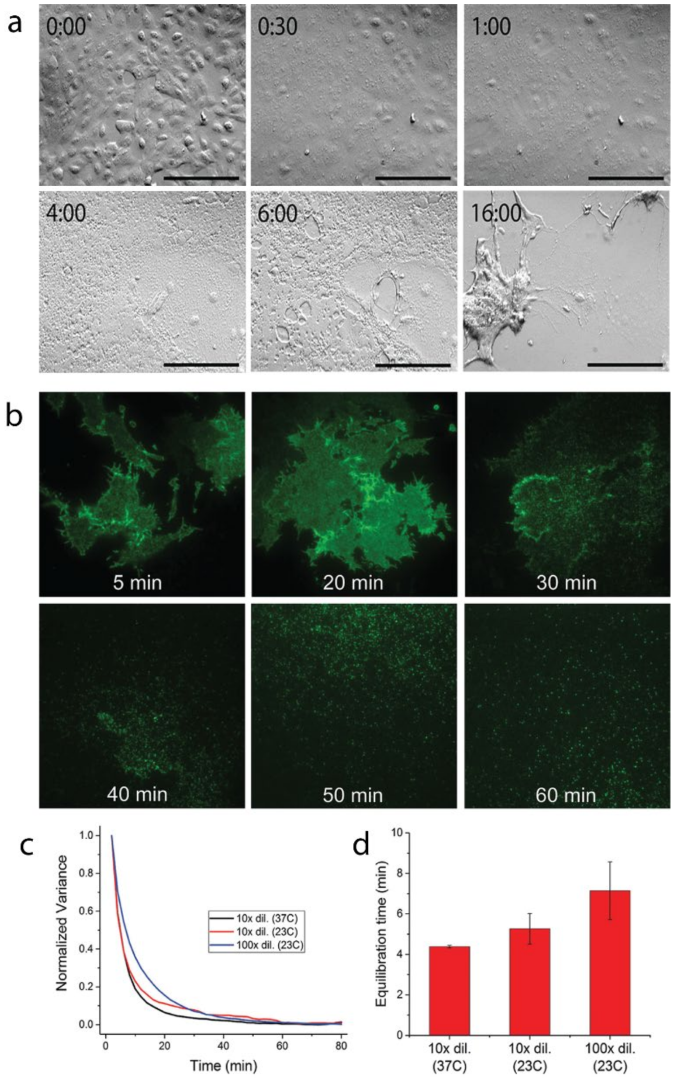

Figure S1: Time course of syncytial formation and protein diffusion in the cytosol and on the membrane.

(a) The cell fusion process acquired using an incubator microscope. Initially each cell's bounding membranes are clearly discernible but this morphology disappears within 30 minutes. The resulting syncytium remains bound to the coverslip for 4-6 hours, at which point it begins to detach from the substrate. By about 16 hours, the syncytium is mostly detached and cells begin to die. Scale bar is 100 μm. (b) mNG-ADRβ2 diffusion in the VSVG-induced syncytium measured by the change in signal variance across the image as fusion precedes. Equilibrium is reached within 30-60 minutes for all conditions (we looked at a number of different co-plating ratios and temperatures). (c) Exponential decay times for image variance in syncytia. Equilibration takes longer for higher co-plating ratios and lower temperatures, as expected for diffusion of macromolecules (mNG-ADRβ2 in this case). (d) Time course of the membrane protein (mNG-ADRβ2) diffusion after cell fusion. Early time points (5 and 20 minutes) show proteins near their original cells. By 30 minutes, single protein complexes are clearly resolved and many fluorescent puncta are visible by 40 minutes.

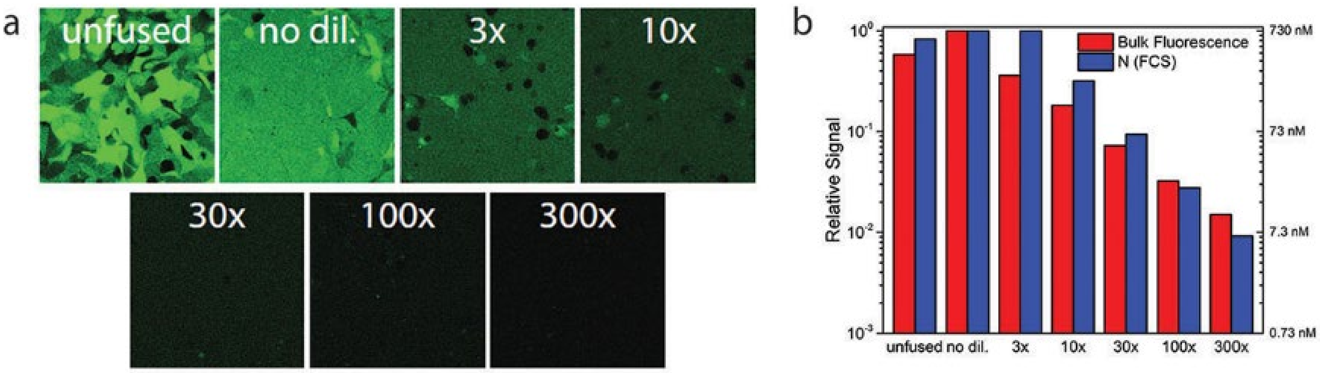

Figure S2: The equilibrium concentration of labeled proteins in syncytia can be set by adjusting the co-plating ratio.

(a) A uniform concentration of labeled proteins can be varied over two orders of magnitude with an upper limit constrained only by the number of cells in the culture dish and the diffusion time of labeled protein complexes compared to syncytium lifetime. We find a 1:10 labelled to VSVG cell ratio produces optimum concentrations for single molecule experiments. (b) Quantification of the data shown in (a) using pixel values (red) and fluorescence correlation spectroscopy (blue). FCS permitted calculation of absolute concentrations.

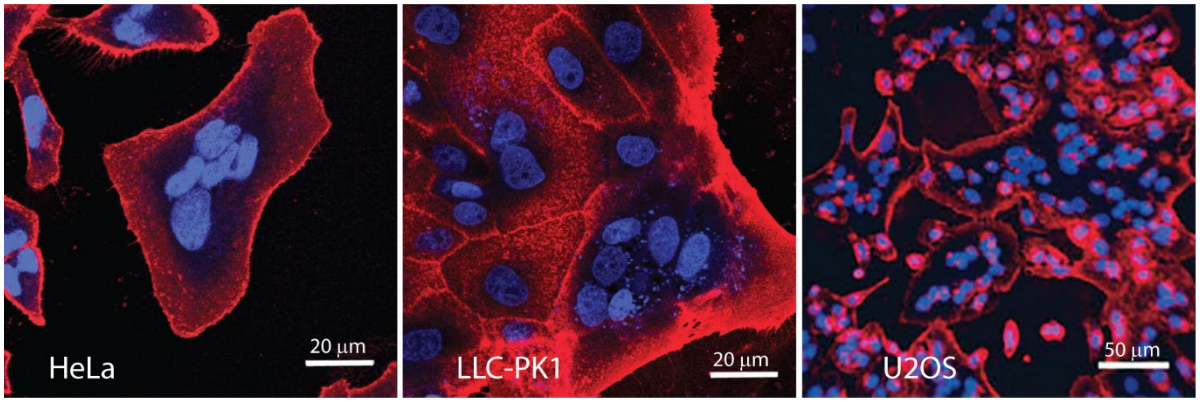

Figure S3: VSVG-mediated fusion in mammalian cell lines

Due to its very broad tropism, VSVG is capable of fusing a large variety of mammalian and non-mammalian cell lines. Here, we demonstrate formation of large syncytia in a human epithelial cancer cell line (HeLa), porcine epithelial cells (LLC-PK1) and Human Bone Osteosarcoma Epithelial Cells (U2OS). Cells were transiently transfected with a constitutive VSVG plasmid (driven by a CMV promoter) and fusion was triggered 24 hours later by a brief incubation in fusion buffer (pH 6.0). After one hour, cells were fixed and membrane and nuclei labeled using Wheat Germ Agglutinin and Hoechst 33342. Large-scale fusion on similar time scales was also observed in a hamster fibroblast line (BHK-21), human embryonic kidney cells (HEK-293) and Rat Basophilic Leukemia cells (RBL-2H3), a mast cell model (data not shown).

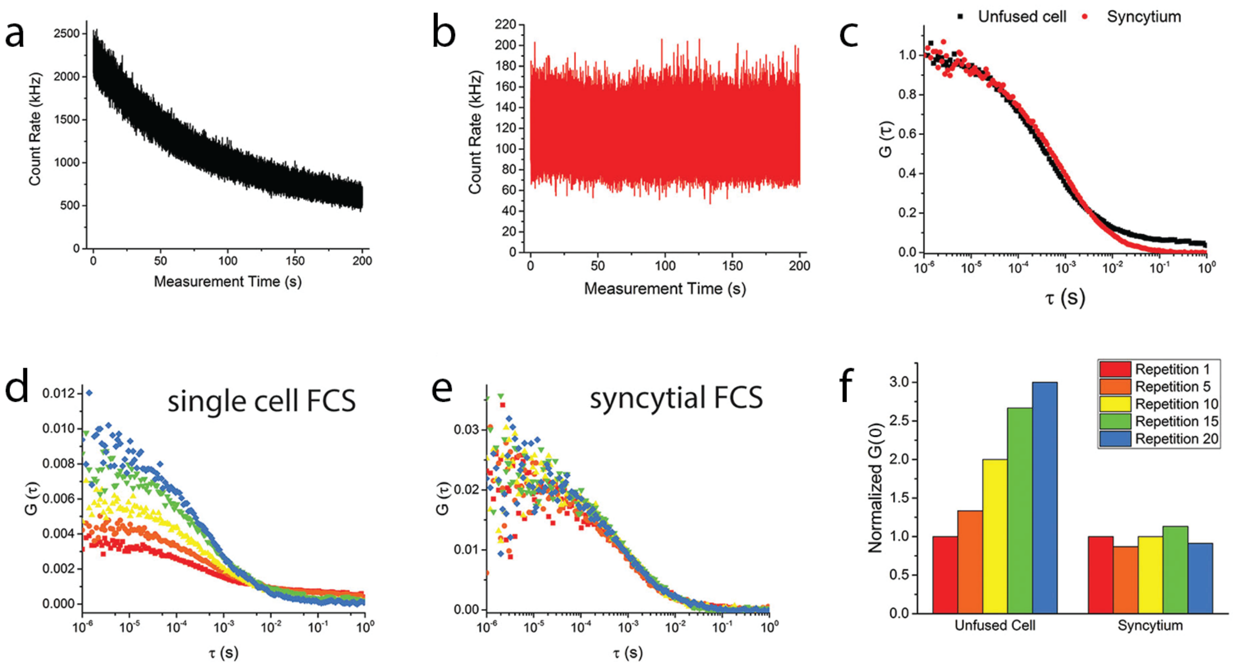

Figure S4: Reduction of live-cell FCS artifacts by the use of SPReAD

(a) Time trace of fluorescence fluctuations in a cell expressing cytoplasmic mNeonGreen. Due to the limited amount of mNeonGreen in the volume of a single cell, the effects of photobleaching can be significant. (b) Photobleaching effects are greatly reduced in large syncytia, where the pool of fluorophore is much larger. (c) Correlation curves for the traces in (a) and (b). The correlation curve for mNeonGreen in syncytia fits well to a 1-component diffusion model, while the single-cell correlation shows a poor fit and does not reach its asymptote as expected (due to photobleaching artifacts). (d) Photobleaching in live cells causes the correlation curve to change over time; G(0) continually rises as fluorescent molecules are depleted. (e) In contrast, syncytial FCS shows no time-dependent changes due to the large pool of freely diffusing labeled proteins. (f) Comparison of G(0) variation over time in live cells and syncytia. G(0) systematically rises in single cells while remaining constant (besides noise) in large syncytia.

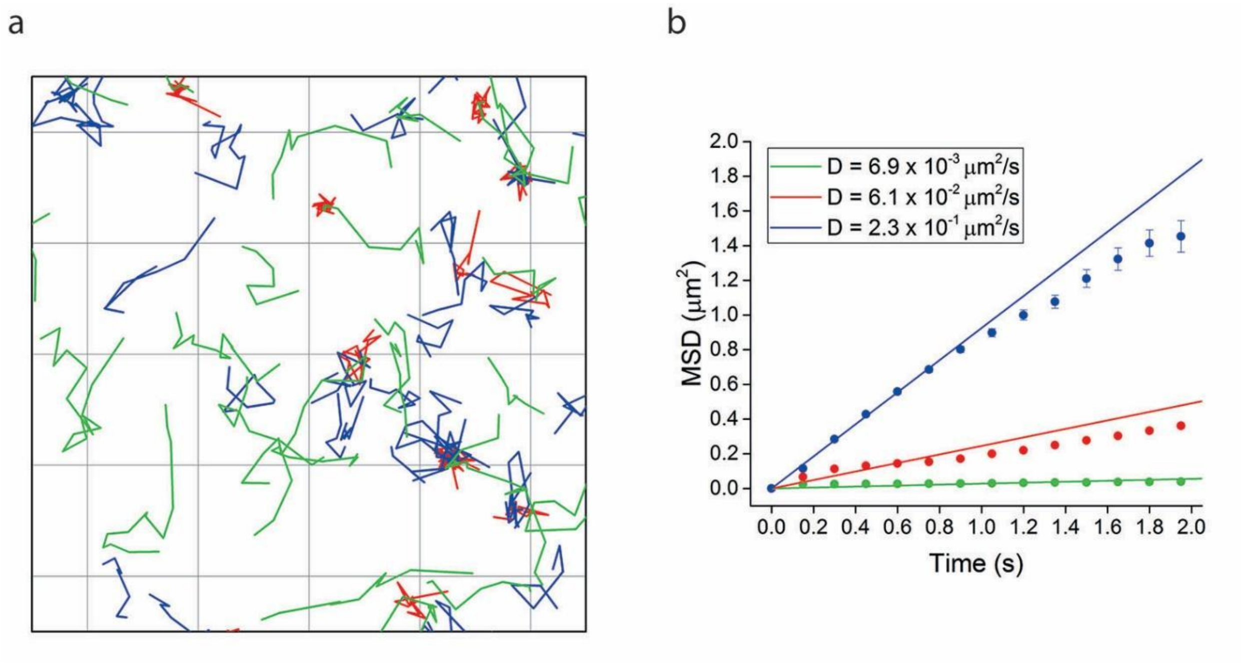

Figure S5: Single-particle tracking of ADRβ2 in large syncytia.

(a) Single particle tracking of mNG-ADRβ2 in a 5 μm square of syncytial membrane. Trajectories were categorized as slow, intermediate and fast based on their diffusion coefficient, Membrane protein diffusion after cell fusion matches previous SPT measurements conducted in live cells, signifying that the syncytial environment preserves the biophysical properties of the plasma membrane. (b) Average MSD plots for slow, intermediate and fast trajectories. Extracted diffusion coefficients are consistent with the range of parameters measured in live cells.

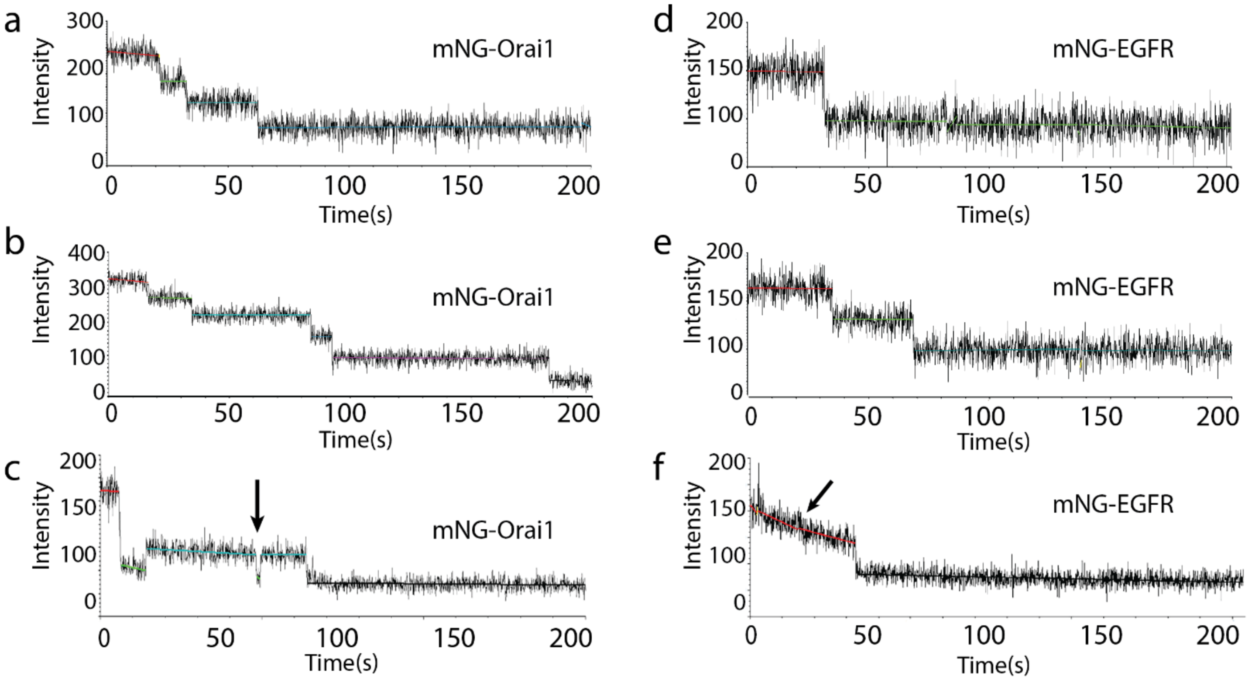

Figure S6: Examples of stepwise photobleaching traces.

(a) – (c) Bleach step traces of mNG-Orai1 complexes: (a) 3 steps, (b) 5 steps, (c) an example of fluorophore blinking artifact (arrow). This trace could be scored as 2 or 3 steps depending on whether drop at around 20s is considered to be background level or the intensity of a single fluorophore. (d) – (f) mNG-EGFR complexes: (d) typical single step trace (EGFR monomer), (e) 2 bleach steps (EGFR dimer), (f) an example of a trace with decaying signal (arrow). Both software used (PIF and our own) corrected for this artifact by fitting the background decay (all pixels not identified as PSFs) and subtracting it from the data before analysis (traces a through e have been adjusted to remove any background decay signal). Intensities are the average of a 5x5 pixel box (500x500 nm) centered on the PSF.

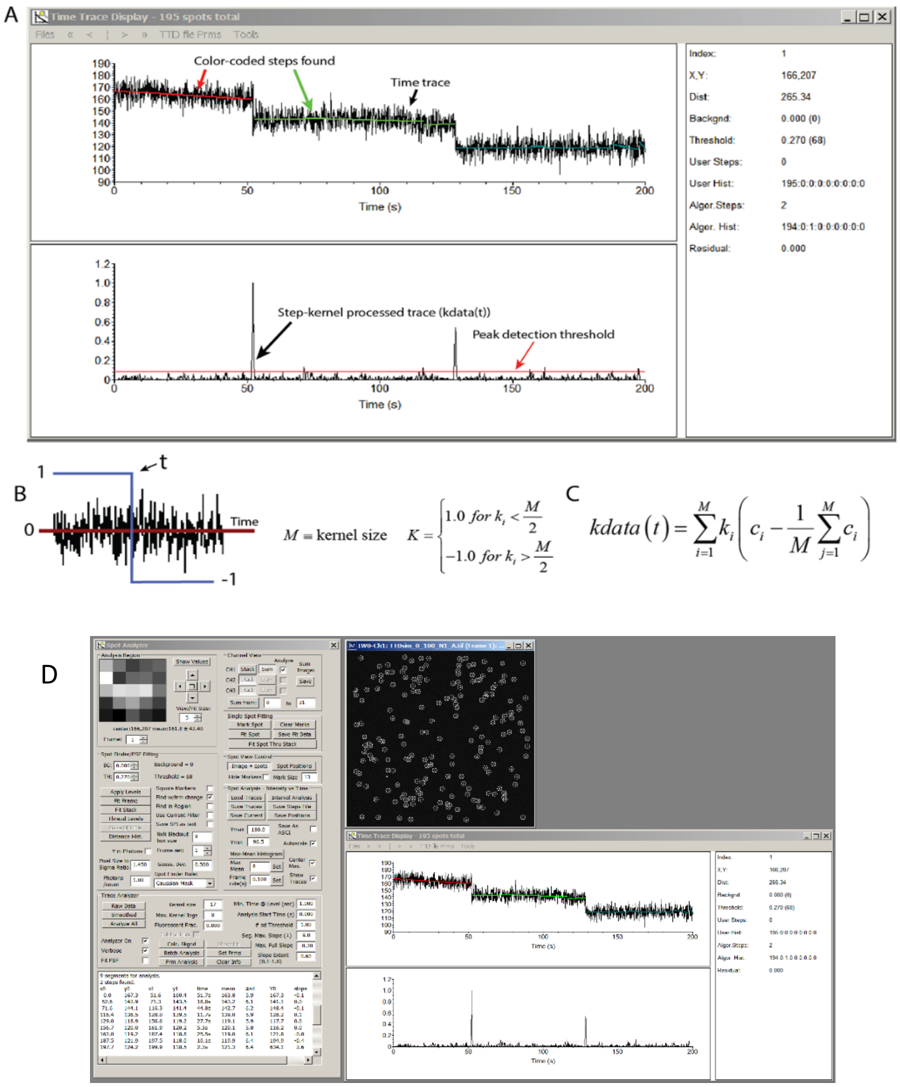

Figure S7: ImageC Analysis software used for bleach step quantification.

The algorithm begins by convoluting the time trace with the kernel defined in B. kdata(t) is effectively the derivative of the time trace. kdata(t) is squared to make it positive, normalized and displayed in the lower plot window of the Time Trace Display window (lower plot in A). The peaks in kdata(t) locate potential bleach steps in time trace as shown above in A. The peak detection threshold level determines how many steps to use in the processing algorithm. Once a peak threshold is found that finds no more than user-set maximum number of allowed “jogs” (convoluted time trace spikes), the time trace is broken up into #jogs + 1 segments defined by the threshold crossing points. The segments are fit to a linear function to obtain their slope. A characteristic deviation (noise level) is calculated for each segment and if the segments are of acceptable length (in time) and slope, they are further analyzed to identify whether they overlap in pixel value. If so, they are considered to be in the same level group (i.e. a fluorophore blinking, rather than a different molecule) rather than assigned as new level. The total number of levels are tallied and the number minus 1 (to account for the final fully bleached level) is reported by ImageC to be the number of photobleach steps in the trace.

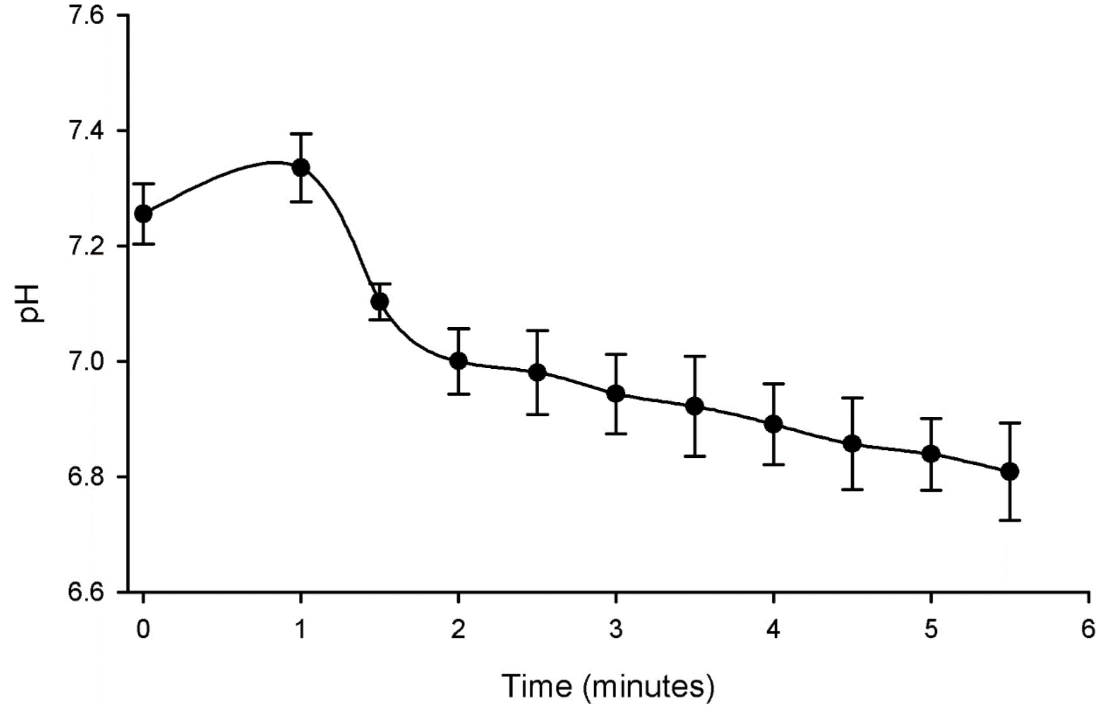

Figure S8: Effect of the short pH drop on intracellular pH using SNARF.

After a 24-hour induction period in doxycycline, Tet-VSVG U2OS cells fuse immediately after a brief pH drop from 7.4 to a pH < 6.0. We measured the effect on the intracellular pH during incubation at pH 5.5 and found that during the short time needed for activating VSVG (0-2 minutes) the pH remains above pH 7.0. Even during longer incubations (up to 5 minutes), the pH was never found to be lower than ~6.8. Data are mean ±SEM n = 2.

mNG-ADRβ2

Number of Steps	#	%	Adjusted %
1	507	72.2	64.3
2	178	25.4	31.8
3	16	2.3	3.6
4	1	0.1	0.3
Sum	702		

mNG-EGFR (non-stimulated)

Number of Steps	#	%	Adjusted %
1	573	67.7	58.5
2	243	28.7	35.9
3	27	3.2	4.6
4	4	0.5	1.0
Sum	847		

mNG-Orai1

Number of Steps	#	%	Adjusted %
1	1572	7.6	0.0
2	3445	21.6	2.2
3	3842	26.1	13.2
4	2993	21.2	22.8
5	1741	13.0	24.6
6	806	6.3	17.9
7	319	2.6	17.1
8	170	1.5	2.2
Sum	14888		

mNG-EGFR (10 min after 200 ng/mL EGF)

Number of Steps	#	%	Adjusted %
1	437	55.3	47.8
2	262	33.2	35.8
3	74	9.4	12.2
4	16	2.1	4.2
Sum	789	100	

mNG-mGluR3 (SPReAD)

Number of Steps	#	%	Adjusted %
1	4816	37.4	27.6
2	4309	33.5	32.5
3	2333	18.1	22.2
4	941	7.3	11.0
5	328	2.5	4.0
6	99	0.8	2.7
Sum	12826		

mNG-mGluR3 (SiMPull)

Number of Steps	#	%	Adjusted %
1	9114	52.5	43.0
2	5377	31.0	33.6
3	1972	11.4	14.9
4	618	3.6	5.7
5	195	1.1	1.2
6	82	0.5	1.6
Sum	17358		

mNG-mGluR5 (SPReAD)

Number of Steps	#	%	Adjusted %
1	4257	36.7	26.7
2	3925	33.9	32.5
3	2178	18.8	22.7
4	877	7.6	12.2
5	267	2.3	3.5
6	82	0.7	2.5
Sum	11586		

mNG-mGluR5 (SiMPull)

Number of Steps	#	%	Adjusted %
1	14151	57.0	49.5
2	6997	28.2	29.9
3	2552	10.3	13.2
4	783	3.2	5.1
5	242	1.0	0.8
6	115	0.5	1.6
Sum	24840		

Table S1: Photobleach step histograms

Frequency of bleach steps for each of the membrane protein oligomers studied. Adjusted percentages are adjusted assuming 20% dark fraction for mNeonGreen.

Supplementary Movies

Video 1: mNG-ADRβ2 mobility after cell fusion (ADRBeta2_MobilityInFusedCells.avi)
Diffusion kinetics of mNeonGreen-tagged ADRβ2 oligomers in syncytial membranes are similar to membrane proteins in unfused cells, suggesting that cell fusion does not significantly perturb the biophysical character of the plasma membrane.

Video 2: Long time course imaging of syncytial formation (FusionTimeCourse_Brightfield.avi)
Oblique-illumination brightfield imaging after pH drop. Cells fuse at beginning of movie and bounding membranes are seen to disappear within 30 minutes. A single syncytium remains bound to the imaging dish for 5-6 hours, after which it begins to detach from the glass coverslip. Detachment progresses until ~20 hours, at which point massive cell death is observed. Fluorescence correlation spectroscopy or formaldehyde fixation (for TIRF experiments) shown is this work were performed during the first 1-2 hours to minimize perturbation of cellular protein complexes.

Video 3: Confocal imaging of cytoplasmic protein diffusion during cell fusion (mNeonGreen_InFusingCells.avi)
Dynamics of mNeonGreen diffusion out of parent cells and into the larger syncytium. Cytoplasmic fluorescent proteins are mobile immediately following pH drop, with equilibrium concentrations being reached within 30-40 minutes (for cytoplasmic mNeonGreen). Equilibration time depends on the molecular weight of the diffusive species, interactions with other cellular components, the ratio of expressing:non-expressing cells and temperature which was 10 to 1 in this case. Field of view is 40 x 40 μm.
